# Supplementary material for: Cigarette Smoking Is Associated with Increased Risk of Malignant Gliomas: A Nationwide Population-Based Cohort Study
Source: Cancers (Basel). 2020 May 25;12(5):1343. doi: 10.3390/cancers12051343 (PMC7280985; doi:10.3390/cancers12051343)
Supplement: Supplementary file 1 [file cancers-12-01343-s001.pdf]

Supplementary Materials

Cigarette Smoking Is Associated with Increased Risk of Malignant Gliomas: A Nationwide Population-Based Cohort Study

Stephen Ahn, Kyung-Do Han, Yong-Moon Park, Jung Min Bae, Sang Uk Kim, Sin-Soo Jeun and Seung Ho Yang

Table S1. Incidence rates and risk of malignant gliomas according to smoking status and age by decade.

Age (Years)	Smoking Status	Total, N	MG Events, n	Person-Years	MG Incidence Rate *
Total	Never-smokers	5,820,623	3,733	42,405,897	8.80
	Former smokers	1,400,124	904	10,143,343	8.91
	Current smokers	2,591,021	1,463	18,731,140	7.81
20-29	Never-smokers	741,153	117	5427663.84	2.16
	Former smokers	92,627	16	681,127	2.35
	Current smokers	392,025	73	2,870,760	2.54
30-39	Never-smokers	835,270	205	6,101,826	3.36
	Former smokers	271,438	81	1,987,193	4.08
	Current smokers	781,384	195	5,700,315	3.42
40-49	Never-smokers	1,503,379	549	11,002,248	4.99
	Former smokers	390,369	157	2,854,917	5.50
	Current smokers	692,445	262	5,033,688	5.20
50-59	Never-smokers	1,307,799	961	9,608,504	10.00
	Former smokers	337,942	222	2,466,245	9.00
	Current smokers	433,503	371	3,130,125	11.85
60-69	Never-smokers	899,820	1046	6,585,467	15.88
	Former smokers	208,538	257	1,498,859	17.15
	Current smokers	207,333	363	1,459,175	24.88
≥ 70	Never-smokers	533,202	855	3,680,189	23.23
	Former smokers	99,210	171	655,002	26.11
	Current smokers	84,331	199	537,078	37.05

CI, confidence interval; HR, hazard ratio; MG, malignant glioma; n, number; * per 100,000 person-years.

© 2020 by the authors. Licensee MDPI, Basel, Switzerland. This article is an open access article distributed under the terms and conditions of the Creative Commons Attribution (CC BY) license (http://creativecommons.org/licenses/by/4.0/).
